# Lower error bounds and optimality of approximation for jump-diffusion SDEs with discontinuous drift

**DOI:** 10.1007/s10543-024-01036-7

**Published:** 2024-09-09

**Authors:** Paweł Przybyłowicz, Verena Schwarz, Michaela Szölgyenyi

**Affiliations:** 1 Faculty of Applied Mathematics, AGH University of Krakow, Al. Mickiewicza 30, 30-059 Krakow, Poland; 2 Department of Statistics, University of Klagenfurt, Universitätsstraße 65-67, 9020 Klagenfurt, Austria

**Keywords:** Jump-diffusion stochastic differential equations, Discontinuous drift, Jump-adapted scheme, Lower bounds, Optimality of approximation schemes, 65C30, 65C20, 60H10, 68Q25

## Abstract

In this paper sharp lower error bounds for numerical methods for jump-diffusion stochastic differential equations (SDEs) with discontinuous drift are proven. The approximation of jump-diffusion SDEs with non-adaptive as well as jump-adapted approximation schemes is studied and lower error bounds of order 3/4 for both classes of approximation schemes are provided. This yields optimality of the transformation-based jump-adapted quasi-Milstein scheme.

## Introduction

Our aim is to provide lower error bounds as well as an optimality result for the approximation of SDEs with discontinuous drift.

For this, we consider the following time-homogeneous jump-diffusion stochastic differential equation (SDE) with additive noise

1.1 $$\begin{aligned} \textrm{d}X_t&= \mu (X_t) \textrm{d}t + \textrm{d}W_t+ \textrm{d}N_t , \quad t\in [0,1], \quad X_0= \xi . \end{aligned}$$

Here $\xi \in {\mathbb {R}}$, $\mu :{\mathbb {R}}\rightarrow {\mathbb {R}}$ is a measurable function, $W=(W_t)_{t\in [0,1]}$ is a Brownian motion, and $(N_t)_{t\in [0,1]}$ is a homogeneous Poisson process with intensity $\lambda \in (0,\infty )$ both defined on a filtered probability space $(\Omega ,{{\mathscr {F}}},\mathbb {F},{\mathbb {P}})$, where $\mathbb {F}=(\mathbb {F}_t)_{t\in [0,1]}$ is a filtration satisfying the usual conditions.

By [1, 2] existence and uniqueness of the solution of the above SDE is well settled under our assumptions.

In the case without jumps upper error bounds for SDEs with discontinuous drift can be found in [3–20] and upper error bounds in the jump-diffusion case are given in [1, 21]. Although [7, 10, 13] consider multiple dimensions and [2] includes jumps in the multidimensional setting, to the best of our knowledge upper error bounds for jump-diffusion SDEs with discontinuous drift in multiple dimensions have not yet been studied. For lower bounds in more regular settings we refer to [22–24]; for lower bounds and optimality results for the approximation of jump-diffusion SDEs with continuous drift, see [25–30]. The first results on lower bounds for scalar jump-free SDEs with discontinuous drift are [31–33]. In [32] convergence order 3/4 is proven to be optimal for non-adaptive approximation schemes based on a finite number of approximations of the driving Brownian motion under the additional assumptions that the drift coefficient is monotonically increasing and bounded. In [33] these additional assumptions are dropped by using the transformation technique introduced in [5, 7, 12]. Further, an extension to SDEs with multiplicative noise based on an application of Lamperti’s transformation is provided.

Our paper adds to the literature in the following aspect: We provide the first lower error bound for the approximation of SDEs with discontinuous drift and in the presence of jumps. We prove bounds for both non-adaptive as well as jump-adapted schemes of order 3/4 in $L^1$ for SDEs with additive noise. The rate 3/4 turns out to be optimal in the case of jump-adapted approximation schemes, since in [21] an upper error bound of the same order is proven in a more general setting for the transformation-based jump-adapted quasi-Milstein scheme.

## Preliminaries

For a Lipschitz continuous function $f$ we denote by $L_f$ its Lipschitz constant. For a vector $v\in {\mathbb {R}}^d$ with $d\in {\mathbb {N}}$ we denote by $v^T$ its transpose. For an integer $d\in {\mathbb {N}}$ and we denote by ${\mathbb {D}}([0,1],{\mathbb {R}}^d)$ the space of all cádlág functions from [0, 1] to ${\mathbb {R}}^d$ equipped with the Skorokhod topology. Denote by $\nu _1, \ldots \nu _{N_1}$ the points of discontinuity of the path of the Poisson process on the interval [0, 1] ordered strictly increasing. Further, denote by $l^1$ the space of all real valued sequences $(x_n)_{n\in {\mathbb {N}}}$ with $\Vert (x_n)_{n\in {\mathbb {N}}}\Vert = \sum _{n=1}^\infty |x_n| <\infty$.

### Assumption 2.1

We assume that the drift coefficient $\mu :{\mathbb {R}}\rightarrow {\mathbb {R}}$ of SDE (1.1) satisfies:

(i) it is piecewise Lipschitz continuous, i.e. there exist $k\in {\mathbb {N}}$ and $-\infty =\zeta _0<\zeta _1<\ldots< \zeta _k <\zeta _{k+1}=\infty$ such that $\mu$ is Lipschitz continuous on $(\zeta _{i}, \zeta _{i+1})$ for every $i\in \{0, \ldots , k\}$.
(ii) it is differentiable with Lipschitz continuous derivative on $(\zeta _{i}, \zeta _{i+1})$ for every $i\in \{0, \ldots , k\}$.
(iii) there exists $i\in \{1,\dots ,k\}$ such that $\mu (\zeta _i+)\not =\mu (\zeta _i-)$.

Under the same assumptions on $\mu$ we introduce the jump-free SDE

2.1 $$\begin{aligned} \begin{aligned} \textrm{d}Y_t = \mu (Y_t)\textrm{d}t + \textrm{d}W_t, \quad t\in [0,1], \quad Y_0 = \xi . \end{aligned} \end{aligned}$$

The existence and uniqueness of the solution *Y* is guaranteed by [5, 7].

For the proof of our main result we will reduce the complexity of our problem to be able to apply the lower bound for the jump-free case [33, Theorem 1]. For this we will need a joint functional representation for the solutions of SDE (1.1) and SDE (2.1).

### Lemma 2.1

Assume that $\mu$ satisfies Assumption 2.1 (i). Then there exists a Skorokod measurable mapping

$$\begin{aligned} \begin{aligned} F:{\mathbb {R}}\times {\mathbb {D}}([0,1],{\mathbb {R}}^3)\rightarrow {\mathbb {D}}([0,1],{\mathbb {R}}) \end{aligned} \end{aligned}$$

such that the unique strong solution of SDE (1.1) is $F(\xi ,(Id,W,N)^T)$ and the unique strong solution of SDE (2.1) is $F(\xi ,(Id,W,0)^T)$.

### Proof

First we recall the transformation form [1], which is a variant of the original construction from [7]. Let $\phi :{\mathbb {R}}\rightarrow {\mathbb {R}}$ be defined by

$$\begin{aligned} \phi (u)= {\left\{ \begin{array}{ll} (1+u)^3(1-u)^3 &  \text {if } |u|\le 1,\\ 0 &  \text {otherwise}. \end{array}\right. } \end{aligned}$$

Based on this we define *G* by

$$\begin{aligned} G(x)=x+ \sum _{j=1}^k \alpha _j\phi \!\left( \frac{x-\zeta _j}{c}\right) (x-\zeta _j)|x-\zeta _j| , \end{aligned}$$

where

$$\begin{aligned} \alpha _j =\frac{\mu (\zeta _j-)-\mu (\zeta _j+)}{2}, \qquad j\in \{1,\dots ,k\} \end{aligned}$$

and

$$\begin{aligned} c\in \bigg (0,\min \bigg \{\min \limits _{1\le j\le k}\frac{1}{6|\alpha _j|},\min \limits _{1\le j\le k-1}\frac{\zeta _{j+1}-\zeta _j}{2}\bigg \}\bigg ), \end{aligned}$$

when we use the convention $1/0=\infty$. This function *G* satisfies the following properties, see [7, Lemma 3.8 and Lemma 2.2]:

- For all $x\in {\mathbb {R}}$ it holds that $G'(x)>0$;
- $G$ has a global inverse $G^{-1}:{\mathbb {R}}\rightarrow {\mathbb {R}}$;
- *G* and $G^{-1}$ are Lipschitz continuous;
- $G\in C^1_b$, which means that *G* is continuously differentiable and has a bounded derivative;
- $G'$ is a Lipschitz continuous function.

Now we apply the transformation *G* to SDE (1.1) and SDE (2.1). For SDE (1.1) we get as in the proof of [1, Theorem 3.1] that $Z=G(X)$ satisfies

$$\begin{aligned} \textrm{d}Z_t={\widetilde{\mu }} (Z_t)\textrm{d}t+{\widetilde{\sigma }}(Z_t)\textrm{d}W_t+{\widetilde{\rho }}(Z_{t-})\textrm{d}N_t, \quad t\in [0,1], \quad Z_0= G(\xi ), \end{aligned}$$

where for all $\xi \in {\mathbb {R}}$

$$\begin{aligned} {\widetilde{\mu }} =\Big (G'\mu + \frac{1}{2} G''\Big )\circ G^{-1}, \quad {\widetilde{\sigma }}=G'\circ G^{-1},\text { and }\quad {\widetilde{\rho }}(x)=G(G^{-1}(x)+1)-x. \end{aligned}$$

It holds that ${\widetilde{\mu }}$, ${\widetilde{\sigma }}$, and ${\widetilde{\rho }}$ are Lipschitz continuous. We can also apply *G* to SDE (2.1) and obtain that ${\widetilde{Z}} = G(Y)$ satisfies

$$\begin{aligned} \textrm{d}{\widetilde{Z}}_t={\widetilde{\mu }} ({\widetilde{Z}}_t)\textrm{d}t+{\widetilde{\sigma }}( {\widetilde{Z}}_t)\textrm{d}W_t, \quad t\in [0,1], \quad {\widetilde{Z}}_0= G(\xi ). \end{aligned}$$

We rewrite these SDEs to

$$\begin{aligned} \begin{aligned} \textrm{d}Z_t&= ({\widetilde{\mu }} (Z_{t-}),{\widetilde{\sigma }}(Z_{t-}),{\widetilde{\rho }}(Z_{t-})) \textrm{d}(t,W_t,N_t)^T, \quad t\in [0,1], \quad Z_0= G(\xi ), \end{aligned} \end{aligned}$$

and

$$\begin{aligned} \begin{aligned} \textrm{d}{\widetilde{Z}}_t&= ({\widetilde{\mu }} (\widetilde{Z}_{t-}),{\widetilde{\sigma }}( {\widetilde{Z}}_{t-}),\widetilde{\rho }({\widetilde{Z}}_{t-})) \textrm{d}(t,W_t,0)^T, \quad t\in [0,1], \quad {\widetilde{Z}}_0= G(\xi ). \end{aligned} \end{aligned}$$

The function $({\widetilde{\mu }},{\widetilde{\sigma }},{\widetilde{\rho }})$ satisfies [34, Assumption 2.1] and hence by the Skorohod measurable universal function representation proven in [34, Theorem 3.1] there exists a function $\Psi :{\mathbb {R}}\times {\mathbb {D}}([0,1],{\mathbb {R}}^3) \rightarrow {\mathbb {D}}([0,1],{\mathbb {R}})$ such that

$$\begin{aligned} \begin{aligned} Z = \Psi (G(\xi ),(t,W_t,N_t)^T_{t\in [0,1]}) \text { and } {\widetilde{Z}} = \Psi (G(\xi ),(t,W_t,0)^T_{t\in [0,1]}). \end{aligned} \end{aligned}$$

Next we apply for all $t\in [0,1]$, $G^{-1}$ to $Z_t$ respectively ${\widetilde{Z}}_t$ to obtain

$$\begin{aligned} (X_t)_{t\in [0,1]} =\Biggl (G^{-1}\Bigl [\Bigl (\Psi (G(\xi ),(t,W_t,N_t)^T_{t\in [0,1]})\Bigr )(t)\Bigr ]\Biggr )_{t\in [0,1]} \end{aligned}$$

and

$$\begin{aligned} (Y_t)_{t\in [0,1]} =\Biggl (G^{-1}\Bigl [\Bigl (\Psi (G(\xi ),(t,W_t,0)^T_{t\in [0,1]})\Bigr )(t)\Bigr ]\Biggr )_{t\in [0,1]}. \end{aligned}$$

Defining

$$\begin{aligned} \begin{aligned} F:{\mathbb {R}}\times {\mathbb {D}}([0,1],{\mathbb {R}}^3) \rightarrow {\mathbb {D}}([0,1],{\mathbb {R}}), \quad F(\xi ,x)(\cdot ) = G^{-1}(\Psi (G(\xi ),x)(\cdot )), \end{aligned} \end{aligned}$$

which is as a concatenation of measurable functions again measurable, proves the claim. $\square$

## Main result

### Theorem 3.1

Assume that $\mu$ satisfies Assumption 2.1. Let $\xi \in {\mathbb {R}}$, let $W:[0,1]\times \Omega \rightarrow {\mathbb {R}}$ be a Brownian motion and $N:[0,1]\times \Omega \rightarrow {\mathbb {R}}$ a Poisson process with intensity $\lambda \in (0,\infty )$, which is independent of *W*. Let $X :[0,1]\times \Omega \rightarrow {\mathbb {R}}$ be the strong solutions of SDE (1.1) on the time-interval [0, 1] with initial value $\xi$, driving Brownian motion *W*, and driving Poisson process *N*. Then there exist $c\in (0,\infty )$ such that for all $n\in {\mathbb {N}}$,

$$\begin{aligned} \begin{aligned}&\inf _{\begin{array}{c} t_1,\dots ,t_n \in [0,1]\\ g :{\mathbb {R}}^n \times l^1 \times {\mathbb {D}}([0,1],{\mathbb {R}}) \rightarrow {\mathbb {R}}\\ \text{ measurable } \\ \end{array}} {\mathbb {E}}\bigl [|X_1-g((W_{t_1}, \ldots , W_{t_n}),(W_{\nu _1},...,W_{\nu _{N_1}},0,...), (N_t)_{t\in [0,1]}))|\bigr ] \ge \frac{c}{n^{3/4}}. \end{aligned} \end{aligned}$$

### Proof

Fix $n\in {\mathbb {N}}$, $t_1,\dots ,t_n \in [0,1]$, and $g :{\mathbb {R}}^n \times l^1 \times {\mathbb {D}}([0,1],{\mathbb {R}}) \rightarrow {\mathbb {R}}$ measurable. In the following we make use of considering only those $\omega \in \Omega$ for which $N_1(\omega )=0$. This implies that *N* has no jump until time 1. Using Lemma 2.1 and observing that $(F({\xi }, (Id,W,0)^T)_t)_{t\in [0,1]}$ is the solution of (2.1) we get

$$\begin{aligned} \begin{aligned}&{\mathbb {E}}\Bigl [\Bigl |X_1-g\Bigl ((W_{t_1}, \ldots , W_{t_n}),(W_{\nu _1},...,W_{\nu _{N_1}},0,...), (N_t)_{t\in [0,1]}\Bigr )\Bigl |\Bigr ]\\&\ge {\mathbb {E}}\bigl [|X_1-g((W_{t_1}, \ldots , W_{t_n}),(W_{\nu _1},...,W_{\nu _{N_1}},0,...), (N_t)_{t\in [0,1]})|\cdot \mathbbm {1}_{\{N_1=0\}}\bigr ]\\&= {\mathbb {E}}\bigl [|F({\xi },(Id,W,N)^T)_1-g((W_{t_1}, \ldots , W_{t_n}),(W_{\nu _1},...,W_{\nu _{N_1}},0,...), (N_t)_{t\in [0,1]})|\\&\quad \quad \quad \quad \quad \quad \quad \quad \quad \quad \quad \quad \quad \quad \quad \quad \quad \quad \quad \quad \quad \quad \quad \quad \quad \quad \quad \quad \qquad \qquad \qquad \qquad \quad \,\,\, \cdot \mathbbm {1}_{\{N_1=0\}}\bigr ]\\&= {\mathbb {E}}\bigl [|F({\xi },(Id,W,0)^T)_1-g((W_{t_1}, \ldots , W_{t_n}),(0,...), (0)_{t\in [0,1]})|\cdot \mathbbm {1}_{\{N_1=0\}}\bigr ]\\&= {\mathbb {E}}\bigl [|F({\xi },(Id,W,0)^T)_1-g((W_{t_1}, \ldots , W_{t_n}),(0,...), (0)_{t\in [0,1]})|\bigr ]{\mathbb {P}}(N_1=0)\\&= {\mathbb {E}}\bigl [|Y_1-g((W_{t_1}, \ldots , W_{t_n}),(0,...), (0)_{t\in [0,1]})|\bigr ]{\mathbb {P}}(N_1=0). \end{aligned} \end{aligned}$$

It holds that ${\mathbb {P}}(N_1=0) = \exp (-\lambda )>0$. By [33, Theorem 1] there exists a constant $c\in (0,\infty )$ such that for all $n\in {\mathbb {N}}$,

$$\begin{aligned} \begin{aligned}&\inf _{\begin{array}{c} t_1,\dots ,t_n \in [0,1]\\ h :{\mathbb {R}}^{n} \rightarrow {\mathbb {R}}\text { measurable} \\ \end{array}} {\mathbb {E}}\bigl [|Y_1-h(W_{t_1}, \ldots , W_{t_n})|\bigr ] \ge \frac{c}{n^{3/4}}. \end{aligned} \end{aligned}$$

Since $g((W_{t_1}, \ldots , W_{t_n}),(0,...), (0)_{t\in [0,1]}))$ can be also interpreted as $h(W_{t_1}, \ldots , W_{t_n})$ for $h:{\mathbb {R}}^n\rightarrow {\mathbb {R}}$, $h(x)=g(x, (0,..),(0)_{t\in [0,1]})$ for all $x\in {\mathbb {R}}^n$ it holds that

$$\begin{aligned} \begin{aligned}&{\mathbb {E}}\bigl [|X_1-g((W_{t_1}, \ldots , W_{t_n}),(W_{\nu _1},...,W_{\nu _{N_1}},0,...), (N_t)_{t\in [0,1]}))|\bigr ]\\&\ge {\mathbb {E}}\bigl [|{Y}_1-g((W_{t_1}, \ldots , W_{t_n}),(0,...), (0)_{t\in [0,1]}))|\big |\bigr ]{\mathbb {P}}(N_1=0)\\&\ge \inf _{\begin{array}{c} t_1,\dots ,t_n \in [0,1]\\ h :{\mathbb {R}}^{n} \rightarrow {\mathbb {R}}\text { measurable} \\ \end{array}} {\mathbb {E}}\bigl [|{Y}_1-h(W_{t_1}, \ldots , W_{t_n})|\bigr ] \cdot \exp (-\lambda ) \ge \exp (-\lambda ) \frac{c}{n^{3/4}}. \end{aligned} \end{aligned}$$

Since this holds for all $n\in {\mathbb {N}}$, $t_1,\dots ,t_n \in [0,1]$, and $g :{\mathbb {R}}^n \times l^{1} \times {\mathbb {D}}([0,1],{\mathbb {R}}) \rightarrow {\mathbb {R}}$ measurable, the claim is proven. $\square$

### Remark 3.1

In the proof of this result we used the knowledge obtained for SDEs without jump noise in [33, Theorem 1] together with the functional representation determined in Lemma 2.1. An extension as in [33, Corollary 1] using simply Lamperti’s transformation is not possible, since transforming the SDE into an SDE with additive diffusion leads to a non-additive jump noise. An extension to multiplicative noise therefore has to be postponed to future research.

In [21, Theorem 4.4] an upper error bound of order 3/4 is proven for the so-called transformation-based jump-adapted quasi-Milstein scheme. In Theorem 3.1 we prove a lower error bound of the same order, which holds for every class of SDEs that contain an SDE (1.1) satisfying Assumption 2.1. Hence, this implies optimality in the worst-case setting of the transformation-based jump-adapted quasi-Milstein scheme for every class of jump-diffusion SDEs that contain a particular equation (1.1) and for which the coefficients satisfy the assumptions of [21, Theorem 4.4]. In particular, we obtain that for SDE (1.1) under Assumption 2.1 the error rate 3/4 is optimal and it is achieved by the transformation based jump-adapted quasi-Milstein scheme.

### Corollary 3.1

Assume that $\mu$ satisfies Assumption (2.1). Let $\xi \in {\mathbb {R}}$, let $W:[0,1]\times \Omega \rightarrow {\mathbb {R}}$ be a Brownian motion and $N:[0,1]\times \Omega \rightarrow {\mathbb {R}}$ a Poisson process with intensity $\lambda \in (0,\infty )$, which is independent of *W*. Let $X :[0,1]\times \Omega \rightarrow {\mathbb {R}}$ be the strong solutions of SDE (1.1) on the time-interval [0, 1] with initial value $\xi$, driving Brownian motion *W*, and driving Poisson process *N*. Further, let $X^{M,n}$ be the transformation based jump-adapted quasi-Milstein scheme based on *n* equidistant grid points for all $n\in {\mathbb {N}}$. Then it holds that

$$\begin{aligned} {\mathbb {E}}\Bigl [\sup _{t\in [0,1]}\big |X_t-X^{M,n}_t\big |\Bigr ] = \Theta (n^{-3/4}). \end{aligned}$$

### Remark 3.2

For the implementation of the transformation-based jump-adapted quasi-Milstein scheme the inverse of the involved transformation is needed. This transformation includes a polynomial of higher order than the one used in the current paper. Therefore, in general a numerical approximation of the inverse is needed, making the scheme implicit in this sense, but still implementable. Note that an implementation of a transformation-based scheme where a numerical inversion of a similar transformation was applied for other reasons has been used in [7].

The following corollary shows that a lower error bound of order 3/4 can also be obtained for non-adaptive approximation schemes.

### Corollary 3.2

Assume that $\mu$ satisfies Assumption 2.1. Let $\xi \in {\mathbb {R}}$, let $W:[0,1]\times \Omega \rightarrow {\mathbb {R}}$ be a Brownian motion and $N:[0,1]\times \Omega \rightarrow {\mathbb {R}}$ a Poisson process with intensity $\lambda \in (0,\infty )$, which is independent of *W*. Let $X :[0,1]\times \Omega \rightarrow {\mathbb {R}}$ be the strong solutions of SDE (1.1) on the time-interval [0, 1] with initial value $\xi$, driving Brownian motion *W*, and driving Poisson process *N*. Then there exist $c\in (0,\infty )$ such that for all $n\in {\mathbb {N}}$,

$$\begin{aligned} \inf _{\begin{array}{c} t_1,\dots ,t_n \in [0,1]\\ g :{\mathbb {R}}^{2n} \rightarrow {\mathbb {R}}\text { measurable} \\ \end{array}} {\mathbb {E}}\bigl [|X_1-g(W_{t_1}, \ldots , W_{t_n},N_{t_1},\ldots N_{t_n})|\bigr ]\ge \frac{c}{n^{3/4}}. \end{aligned}$$

### Remark 3.3

Corollary 3.2 does not imply any optimality results, since there is still a gap between the upper and lower bounds known from literature. The best upper bounds are provided in [1]. There it is proven that the Euler–Maruyama scheme has convergence order 1/2 in $L^2$, which implies the same rate in $L^1$.

### Conjecture 3.1

For SDE (1.1) satisfying Assumptions 2.1 the convergence rate 1/2 is optimal in $L^2$ for non-adaptive algorithms, which evaluate *W*, *N* at fixed time points.
